# A Systematic Review of the Effects of Hyperoxia in Acutely Ill Patients: Should We Aim for Less?

**DOI:** 10.1155/2018/7841295

**Published:** 2018-05-14

**Authors:** R. Stolmeijer, H. R. Bouma, J. G. Zijlstra, A. M. Drost-de Klerck, J. C. ter Maaten, J. J. M. Ligtenberg

**Affiliations:** ^1^Department of Emergency Medicine, Medical Center Leeuwarden, Leeuwarden, Netherlands; ^2^Department of Clinical Pharmacy & Pharmacology, University Medical Center Groningen, University of Groningen, Groningen, Netherlands; ^3^Department of Internal Medicine, University Medical Center Groningen, University of Groningen, Groningen, Netherlands; ^4^Department of Critical Care, University Medical Center Groningen, University of Groningen, Groningen, Netherlands; ^5^Department of Emergency Medicine, University Medical Center Groningen, University of Groningen, Groningen, Netherlands

## Abstract

**Introduction:**

Despite widespread and liberal use of oxygen supplementation, guidelines about rational use of oxygen are scarce. Recent data demonstrates that current protocols lead to hyperoxemia in the majority of the patients and most health care professionals are not aware of the negative effects of hyperoxemia.

**Method:**

To investigate the effects of hyperoxemia in acutely ill patients on clinically relevant outcomes, such as neurological and functional status as well as mortality, we performed a literature review using Medline (PubMed) and Embase. We used the following terms: hyperoxemia OR hyperoxemia OR [“oxygen inhalation therapy” AND (mortality OR death OR outcome OR survival)] OR [oxygen AND (mortality OR death OR outcome OR survival)]. Original studies about the clinical effects of hyperoxemia in adult patients suffering from acute or emergency illnesses were included.

**Results:**

37 articles were included, of which 31 could be divided into four large groups: cardiac arrest, traumatic brain injury (TBI), stroke, and sepsis. Although a single study demonstrated a transient protective effect of hyperoxemia after TBI, other studies revealed higher mortality rates after cardiac arrest, stroke, and TBI treated with oxygen supplementation leading to hyperoxemia. Approximately half of the studies showed no association between hyperoxemia and clinically relevant outcomes.

**Conclusion:**

Liberal oxygen therapy leads to hyperoxemia in a majority of patients and hyperoxemia may negatively affect survival after acute illness. As a clinical consequence, aiming for normoxemia may limit negative effects of hyperoxemia in patients with acute illness.

## 1. Introduction

Oxygen is a vital element and toxicity may not be immediately obvious. Moreover, impaired oxygen delivery in critically ill patients is associated with increased mortality. As a consequence, reassuring oxygen delivery has become a cornerstone of many resuscitation protocols and liberal use of supplemental oxygen is common [1]. The negative effects of too much oxygen are less clear and many health care professionals are unaware of the possible damage hyperoxemia can cause [2]. In contrast to guidelines describing rational use of drugs, similar guidelines for optimal use of oxygen are scarce. However, the British Thoracic Society (BTS) guideline of 2008 [3] does recommend to aim for (near) normal oxygen saturation for all acutely ill patients and to preclude hyperoxemia. Titration of oxygen therapy appears to be feasible, both in the emergency department (ED) and in the intensive care unit (ICU) [4, 5]. In critical situations, however, oxygen supplementation is generally started without checking for hypoxemia. It is also often not titrated to lower level of oxygen supplementation, despite oxygen saturation readings of (close to) 100% or high partial oxygen pressures (PaO_2_) [6]. Importantly, recent studies have revealed that not only hypoxemia but also hyperoxemia is associated with increased mortality, although data are inconsistent [1, 7–9].

Harmful effects of hyperoxemia may be due to various mechanisms, ranging from vasoconstriction and microvascular blood flow heterogeneity to increased formation of reactive oxygen species (ROS) [1, 10]. Oxygen is a critical element to fuel oxidative phosphorylation for the generation of ATP by mitochondria. Since ATP is mainly produced by means of oxidative phosphorylation, hypoxemia may impair the production of ATP and thereby lead to cellular ATP depletion. On the other hand, hyperoxemia may also lead to mitochondrial dysfunction and depletion of cellular ATP levels. Mitochondria are the major source of ROS, which are formed by reduction of oxygen in the electron transport chain (ETC) [11]. Oxidative damage to the ETC-complexes and inhibition of the citric acid cycle by ROS impedes mitochondrial ATP production, which may impair (active) ion transport and thereby leads to loss of cellular homeostasis [12, 13]. Dysfunction of the Na^+^/K^+^-ATPase can initiate cell membrane depolarization and trigger influx of calcium into the cell through voltage-gated channels [14], which subsequently leads to calcium influx into mitochondria. Although high levels of calcium in the mitochondrial matrix initially increase respiratory rate and ATP production [15], a prolonged rise in calcium within mitochondria induces release of cytochrome *c* and subsequent activation of apoptotic pathways [16]. Taken together, although hypoxemia may impair the generation of ATP, hyperoxemia can also lead to depletion of cellular ATP levels. This is why the standard procedure to supply extra oxygen may lead to mitochondrial damage by increasing the formation of ROS [17]. Moreover, hyperoxemia can result in peripheral vasoconstriction, coronary vasoconstriction, and a decrease in cardiac output [18]. Thus both molecular and physiological effects of (high levels of) oxygen may counteract the positive effects of oxygen supplementation.

Recently, we performed a prospective study in 83 spontaneously breathing patients in the ED to evaluate the effect of conservative oxygen supplementation on blood oxygenation in sepsis. We demonstrated that reducing the inspired oxygen fraction (FiO_2_) from 0.6–0.8 to 0.4 precludes hypoxemia (PaO2 < 9.5 kPa, 9.4 kPa) in 93% of the patients, while 64% are still hyperoxemic (PaO2 > 13.5 kPa, 13.4 kPa) [19]. So even lower levels of oxygen supplementation than prescribed lead to hyperoxemia in the majority of patients. Oxygen is a vital and potentially life-saving element in emergency care, which has led to widespread and liberal oxygen supplementation. But it is important to realize that hyperoxemia is associated with increased mortality and unfavorable outcome, such as more neurological impairment and Acute Respiratory Distress Syndrome (ARDS). Decisions about the initiation and discontinuation of oxygen supplementation in acutely ill patients are made in the ED. However, the optimal and nontoxic level of oxygen supplementation in acutely ill patients is not clear. To this end, we performed a systematic review to assess the association between hyperoxemia in acutely ill patients in the ED and outcome in terms of increased morbidity and mortality.

## 2. Methods

### 2.1. Search Strategy

**Figure 1:**
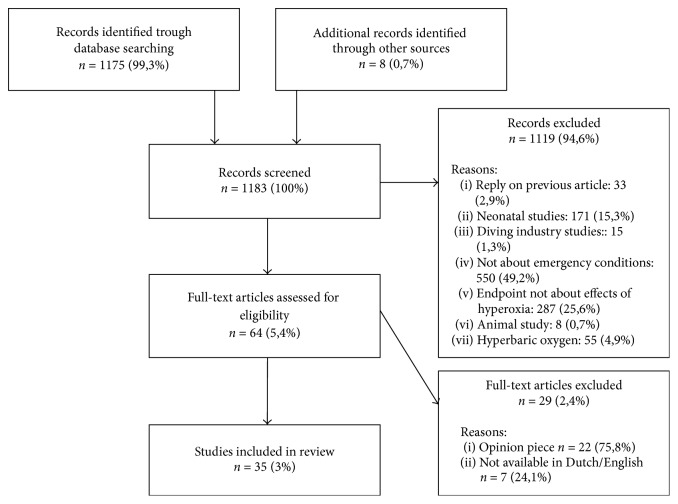
Search results.

We performed a systematic review and searched in databases Medline (PubMed) and Embase on the following terms: hyperoxemia OR hyperoxemia OR [“oxygen inhalation therapy” AND (mortality OR death OR outcome OR survival)] OR [oxygen AND (mortality OR death OR outcome OR survival)]. We searched for studies performed in humans, with full text in English or Dutch available (since these are the languages we are able to read) and that are published in the last 10 years. This 10-year limitation was chosen because of the new insights into hyperoxia. All abstracts were read (TS); if the abstract met an exclusion criterion, the corresponding article was ruled out. This was confirmed by another author (JL). Of the remaining articles, the entire text was read (TS, JL). Of the articles that were included in this way, reference lists were checked for additional relevant publications, which could also be included if they agreed with inclusion and exclusion criteria. This resulted in 8 more suitable articles for inclusion (Figure 1).

### 2.2. Inclusion and Exclusion Criteria

Original studies about the clinical effects of hyperoxemia in adult patients suffering from acute or emergency illnesses were included. Thereby, articles not based on original data (e.g., reviews and comments on previous articles of expert opinion) were not included in the review. We excluded articles (1) not concerning acute conditions and (2) not including clinically relevant effects (e.g., effects on cell level). Most articles could be ruled out by reading the title and abstract, for example, studies performed after birth in neonates, in healthy volunteers, or in the diving industry. Also papers in patients with chronic conditions and about use of hyperbaric oxygen therapy were excluded (Figure 1).

### 2.3. Data Extraction and Analysis

The entire text of all included articles was read and its study design, sample size, definition of hyperoxemia, condition of the patients included, the location of the study, and its main conclusions were summarized in a tabular fashion. Thereby a comprehensive data summary of all included articles was made, which allowed for careful analysis and a comprehensive review of the literature. Differences in study design, heterogeneity, and the different definitions of hyperoxemia and primary outcomes employed in the identified studies lead to a relatively high risk of bias, as assessed using the Cochrane Collaboration's tool. The profound risk of bias hampers performing a meta-analysis. We did not confirm with the authors of the articles that we used. No review protocol exists.

## 3. Results

Our literature search identified 35 manuscripts describing the association between hyperoxemia and clinically relevant outcomes in acutely ill patients. The most important outcomes are mortality, in-hospital mortality, survival, neurological outcome, and organ function. Of the 35 articles, 31 could be divided into four large groups: cardiac arrest, stroke, traumatic brain injury (TBI), and sepsis (Tables 1, 2, and 3). These studies are comprised of four randomized controlled trials (RCT), nine prospective observational studies, and 24 retrospective observational studies. Three other studies were performed in ventilated patients in the ICU, of which 1 study was a RCT. There was one article about patients with a ST-elevated myocardial infarction (STEMI). This article will be discussed first.

Ranchord et al. found no benefit or harm from high-concentration oxygen therapy versus oxygen therapy titrated to normoxia in patients with a STEMI 6 hours after presentation on mortality or infarct size measured by troponin levels [20]. This study was a randomized study performed in 136 patients. Unfortunately the authors do not mention the achieved oxygen values in both groups.

### 3.1. ICU Patients

In one large single-center RCT among 480 ICU patients [50], subjects were randomized either to conservative oxygen supplementation (PaO_2_ 70–100 mmHg or oxygen saturations 94–98%) or to conventional oxygen supplementation (PaO_2_ 100–150 mmHg or oxygen saturations 97–100%). Patients in the conservative group had lower mortality rates, despite early termination of the study due to lower than expected inclusion rates. Eastwood et al. (2012) performed a retrospective cohort study in 152.680 mechanically ventilated patients in 150 ICUs [7]; 49.8% had hyperoxia (PaO_2_ > 16 kPa). An association was found between hypoxia and increased in-hospital mortality, but not between hyperoxia in the first 24 hours and mortality. Another retrospective cohort study performed in mechanically ventilated ICU patients did show an increase in mortality in case of hyperoxia and also in case of hypoxia [9]. In the three ICU studies mentioned in this section, patients formed a heterogeneous group, since they were suffering from all types of diseases.

### 3.2. Cardiac Arrest

Thirteen studies evaluated the potential negative effects of hyperoxemia on neurological outcome and mortality in patients after cardiac arrest, as summarized in Table 1. Hyperoxemia during out-of-hospital cardiopulmonary resuscitation (CPR), measured by point-of-care analysis in the emergency response car, was associated with increased hospital admission rates, as demonstrated in a retrospective cohort study of 1015 patients. No difference in neurologically intact survival was found related to the oxygen concentration, however [21]. Furthermore, the occurrence of hyperoxemia during the first 24 hours of ICU stay in survivors of cardiac arrest is not associated with poorer neurological outcome or increased mortality as compared to normoxemia, as demonstrated in a prospective observational cohort study including 409 patients with 12-month follow-up [22]. Similar to this, no association between the occurrence of hyperoxemia during the first 24 hours after cardiac arrest and in-hospital mortality could be demonstrated in a retrospective cohort study in 584 patients after out-of-hospital cardiac arrest (OHCA) due to ventricular fibrillation (VF) [23], in a retrospective cohort study in 5,258 ICU patients after OHCA [24] and in a larger retrospective cohort study comprising 12,108 patients after cardiac arrest [25]. Hence, different prospective and retrospective observational cohort studies were not able to reveal an association between hyperoxemia during CPR or the first 24 hours after cardiac arrest and poor outcome.

In contrast to these findings, one retrospective cohort study found that early hyperoxemia (during the first 60 minutes after return of spontaneously circulation) in patients with OHCA was associated with better survival rates [26]. Yet in another study both hyperoxemia and hypoxemia are associated with poor neurological outcome, although no influence of hyperoxemia on in-hospital mortality was found [27]. And another retrospective cohort study comprising 6,326 patients after cardiac arrest reveals an independent association between hyperoxemia and higher in-hospital mortality rates, as compared to normoxemia and hypoxemia [28]. In particular, severe hyperoxemia, which is defined as PaO_2_ > 39.9 kPa, seems to be independently associated with increased in-hospital mortality [29]. Moreover, a multicenter study including 4,459 patients after cardiac arrest revealed a linear, dose-dependent association between oxygen and in-hospital mortality: a 13.3 kPa increase in PaO_2_ was associated with a 24% increase in mortality risk [30]. The occurrence of hyperoxemia during postresuscitation care combined with mild hypothermia is independently associated poorer neurological outcome at hospital discharge and increased in-hospital mortality, as demonstrated in an observational cohort study comprising 170 patients. As such, survivors (45%) had a lower maximum PaO_2_ (26.4 kPa) in the first 24 hours versus nonsurvivors (55%; 33.9 kPa) [31]. So in contrast to the lack of an association between (mild) hyperoxemia during the early stages after cardiac arrest and neurologically intact survival, severe hyperoxemia and hyperoxemia during postresuscitation care seem to be associated with poorer outcome.

Current evidence describing the advantages and drawbacks of hyperoxemia during and after CPR is limited to observational studies. Unfortunately, a randomized multicenter single-blind trial (HOT or NOT, 2014), in which the goal was to assess the prehospital effect of oxygen titration versus standard oxygen therapy after out-of-hospital cardiac arrest, was terminated early because titration of oxygen in the prehospital period following OHCA appeared to be unfeasible. The authors suggested that it may be more practicable to titrate the dosage of oxygen down immediately after arrival at the ED or elsewhere in hospital [32]. In conclusion, the level of evidence describing the effect of hyperoxemia on clinically relevant outcome after cardiac arrest is low. Outcomes of studies evaluating hyperoxemia after cardiac arrest are unequivocal, although current data suggests an association between (severe) hyperoxemia during postresuscitation care and lower levels of intact neurological survival.

### 3.3. Stroke

The use of hyperoxemia in acute neurological events is controversial. While some state that it has beneficial effects on the injured brain and should be used as therapy due to its hemodynamic effects, others state that hyperoxemia should be avoided as it may increase neurological damage due to the formation of ROS. We included five studies in patients with stroke (Table 2). The application of hyperoxemia in ventilated patients early after acute ischemic stroke is not associated with poor functional outcome or increased mortality, as revealed in a retrospective study in 2,643 patients [33]. In contrast, a retrospective multicenter study in 2,894 ventilated patients after acute ischemic stroke, subarachnoid hemorrhage (SAH), and intracerebral hemorrhage demonstrated an independent association between hyperoxemia and increased in-hospital mortality. This was 60% after hyperoxemia and 47% after normoxemia [34]. The effect of hyperoxemia on neurological outcome was not assessed in this study unfortunately. Another retrospective cohort study in mechanically ventilated patients with a SAH in the ICU showed that patients with unfavorable outcome (Glasgow Coma Score 1–3) had significantly higher PaO_2_ levels. But in a multivariate regression analysis no association between PaO_2_ and unfavorable outcome of mortality was found [35]. A small randomized and partially blinded pilot study in which patients were treated with high levels of oxygen supplementation (45 L/min for 8 hours) or ambient air after acute ischemic stroke revealed a transient improvement of clinical deficits and MRI abnormalities at 24 hours after inclusion. This disappeared at three-month follow-up [36]. In this study the effect of hyperoxemia on mortality was not assessed. Another randomized study where 40 patients with acute ischemic stroke received either high oxygen supplementation (10 L/min for 12 hours) or ambient air was not able to demonstrate an association between high oxygen supplementation and neurological or functional outcome during three-month follow-up [37]. In summary, the association between hyperoxemia after stroke has been described in a very low number of studies, who either describe no effect of hyperoxemia on clinically relevant outcomes or suggest minor transient protective effects of hyperoxemia.

### 3.4. Traumatic Brain Injury

Hyperoxemia may have protective effects after TBI by improving brain oxygenation and thereby preventing ischemic injury. The application of hyperoxemia after TBI improves brain tissue oxygenation indeed, as demonstrated by cerebral microdialysis, brain tissue oximetry, and oxygen-15 positron emission tomography (PET) in a small study comprising 11 patients with TBI [38]. The use of hyperoxemia not only increases brain oxygen levels, but subsequently also leads to a reduced lactate-pyruvate ratio, which suggests an improved preservation of mitochondrial respiration [39, 40]. Despite an improvement in brain oxygenation and metabolism after TBI, a smaller study comprising five patients with TBI demonstrated no effects on arterial blood pressure, intracranial pressure, and cerebral blood flow when patients were subjected to a FiO2 of 0.3–0.5 or 1.0 for one hour [41]. Moreover, the use of hyperoxemia in TBI patients is not associated with increased levels of oxidative stress or changes in antioxidant reserves in spinal fluid [42]. Hence, hyperoxemia is associated with improved oxygenation and better mitochondrial respiration after TBI.

The occurrence of hyperoxemia after severe TBI is associated with a decrease in good clinical outcome (such as being able to be discharged home) and higher levels of mortality, as demonstrated in a registry based retrospective cohort comprising 3,420 patients with severe TBI [43]. Similar findings were obtained in a multicenter retrospective registry study among 1,212 mechanically ventilated patients suffering from TBI, where hyperoxemia within 24 hours after admission to the ICU is independently associated with higher in-hospital mortality rates [44]. These findings are in line with a small retrospective cohort study, demonstrating increased mortality among patients with either hypoxemia (PaO_2_< 33.3 kPa) and hyperoxemia (PaO_2_> 64.8 kPa) as compared to normoxemia after TBI [45]. Another relatively large retrospective cohort study, comprising 1,116 patients with TBI, was not able to reveal an association between hyperoxemia (> 13.3 kPa) and mortality at six-month follow-up in contrast [46]. In conclusion, although hyperoxemia after TBI improves brain oxygenation and mitochondrial function, the use of hyperoxemia seems to be associated with increased in-hospital mortality.

### 3.5. Sepsis

In contrast to cardiac arrest, stroke, and TBI, optimizing cellular oxygen delivery may be even more difficult in patients with sepsis, which is characterized by a reduced cellular oxygen extraction from the circulation. This phenomenon called “*cytopathic hypoxia*” is reflected in the relatively high venous oxygen level as compared to the arterial oxygen level and is likely due to mitochondrial dysfunction [51–53]. Mitochondrial dysfunction is an early and important event that may progress into loss of cellular homeostasis, organ failure, and ultimately death of the patient [54, 55]. The induction of brief hyperoxemia, by increasing the FiO_2_ to 1.0 for 20 minutes in ventilated patients with severe sepsis and septic shock, paradoxically even decreases oxygen delivery in the upper limbs [47]. High central venous oxygen levels (ScvO_2_; 90–100%) are associated with increased mortality rates, as demonstrated in a prospective study comprising 619 septic patients treated with early goal-directed therapy in the ED [48]. It should be noted, however, that the ScvO_2_ during sepsis is not only influenced by the level of oxygen supplementation, but also by the occurrence of cytopathic hypoxia, which leads to a rise in ScvO_2_. Therefore, arterial oxygen levels better reflect the effect of oxygen supplementation in sepsis. High levels of oxygen supplementation (reflected by FiO_2_ > 60%) and also hypoxemia (PaO_2_ < 8 kPa) are associated with higher in-hospital mortality rates among 1,770 patients with severe sepsis or septic shock admitted to the ICU [49].

## 4. Discussion

Despite the longstanding and ubiquitous use of oxygen there is a paucity of data regarding its optimal use. Remarkably, guidelines describing the optimal dose of oxygen to be supplemented are scarce and current evidence is largely of low level, since it is largely based on observational cohort studies. There are only a few randomized prospective studies describing the effects of hyperoxemia on clinically relevant outcomes. Although we aimed to review the described effects of hyperoxemia on clinically relevant outcomes among acutely ill patients at the ED, most studies describe the effects of hyperoxemia after cardiac arrest, stroke, TBI, or sepsis among patients admitted to the ICU. However, since most patients presenting with these conditions will be primarily admitted to the ED before going to the ICU, we feel that these presented findings can also be applied to these specific ED populations. Yet it remains to be studied whether a similar association between hyperoxemia and clinically relevant outcomes (such as neurological or functional status and mortality) also applies to the patients at the ED that will not be admitted to the ICU. Investigating the potentially negative effects of hyperoxemia in this population may be of major clinical relevance, since oxygen treatment is mostly initiated in the ambulance and continued or started in the ED. There is the need for, preferably randomized, trials with well-defined outcome parameters, including neurological outcome, quality of life, and mortality, to achieve a higher level of evidence.

## 5. Conclusion

Despite the widespread and liberal use of oxygen supplementation in patients with acute illness, which is expected to lead to hyperoxemia in the majority of patients, studies describing the association between hyperoxemia and clinically relevant outcomes are scarce. Although a single study suggested transient improvement of clinical deficits after ischemic stroke treated with high oxygen supplementation, all other studies reviewed here revealed no positive association between hyperoxemia and outcome (such as neurological or functional recovery and mortality). Importantly, several studies described a clear association between hyperoxemia and increased mortality after cardiac arrest, stroke, and TBI. The association between hyperoxemia in sepsis and outcome remains to be studied. Despite these uncertainties, measuring blood oxygenation and aiming for normoxemia may potentially lower the morbidity and mortality associated with hyperoxemia. There is some support that the old paradigm by Paracelsus that toxicity only depends on dose is also true for the highly valued oxygen.

## Figures and Tables

**Table 1:** Association between hyperoxemia and clinically relevant outcomes after myocardial infarction and cardiac arrest.

Reference	Study design	Sample size	Hyperoxemia definition	Condition	Location	Conclusion
[20], Ranchordet al.	RCT	136	6 L O_2_/min	STEMI	-	High-O_2_ therapy had no effect on mortality or infarct size

[21], Spindelboeck et al.	Retrospective cohort	1015	PaO_2_> 40.0 kPa	Cardiac arrest	Pre-hospital	Higher hospital admission rates when during CPR

[22], Vaahersalo et al.	Prospective cohort	409	PaO_2_> 40.0 kPa	Cardiac arrest	ICU	No association with different 12 month outcome

[23], Ihle et al.	Retrospective cohort	584	PaO_2_> 40.0 kPa	Cardiac arrest	ICU	No association with in-hospital mortality

[24], Helmerhorst et al.	Retrospective cohort	5258	PaO_2_ > 39.9 kPa	Cardiac arrest	ICU	Hyperoxia not associated with higher mortality rates

[25], Bellomo et al.	Retrospective cohort	12,108	PaO_2_> 40.0 kPa	Cardiac arrest	ICU	No association with mortality

[26], Chirst et al.	Retrospective cohort	134	-	Cardiac arrest	-	Hyperoxia in the first 60 minutes after return of circulation is associated with better survival rates

[27], Lee et al.	Retrospective cohort	213	-	Cardiac arrest	-	Hypocarbia associated with in-hospital mortality. Hypoxemia and hyperoxemia associated with poor neurological outcome.

[28], Kilgannon et al.	Retrospective cohort	6,326	PaO_2_> 40.0 kPa	Cardiac arrest	ICU	Higher mortality rates, even when compared to hypoxemia

[29], Elmer et al.	Retrospective analysis of prospective registry	184	Severe: PaO_2_> 40.0 kPa Moderate/ probable: PaO_2_ 13.5–39.9 kPa	Cardiac arrest	ICU	Severe associated with higher in-hospital mortality. Moderate/probable was not but was associated with improved organ function after 24 hours.

[30], Kilgannon et al.	Retrospective cohort	4,459	-	Cardiac arrest	ICU	Dose-dependent association with in-hospital mortality

[31], Janz et al.	Post-hoc analysis of prospective cohort	170	-	Cardiac arrest	Cardiovascular care unit	Higher in-hospital mortality and poor neurological status on hospital discharge in survivors

[32], Young et al.	RCT	18	-	Cardiac arrest	Prehospital	Study terminated early, because pre-hospital oxygen titration was not feasible.

**Table 2:** Association between hyperoxemia and clinically relevant outcomes after stroke and traumatic brain injury.

Reference	Study design	Sample size	Hyperoxemia definition	Condition	Location	Conclusion
[33], Young et al.	Retrospective cohort	2,643	-	Ischaemic stroke	ICU	No association with mortality.

[34], Rincon et al.	Retrospective cohort	2,894	PaO_2_> 40.0 kPa	Ischaemic stroke, subarachnoid or intracerebral hemorrhage	ICU	Associated with higher in-hospital mortality, also when compared to hypoxemia.

[35], Lång et al.	Retrospective cohort	432	-	Subarachnoidal hemorrhage	ICU	Unfavorable outcome associated with higher PaO_2_, but higher PaO_2_ levels after multivariate analysis not associated with unfavorable outcome or mortality

[36], Singhal et al.	Randomized pilot study, partially blinded	16	O_2_ 45 L/min, 8 hours	Ischaemic stroke	-	Transient improvement of clinical deficits and MRI abnormalities after 24 hours

[37], Padma et al.	Randomized pilot study, partially blinded	40	O_2_ 10 L/min, 12 hours	Ischaemic stroke	-	No improvement in functional or neurological outcome after 3 months

[38], Nortje et al.	Prospective cohort	11	FiO_2_ 35–50%	TBI	-	Increases brain tissue oxygenation.

[39], Tisdall et al.	Prospective cohort	8	FiO_2_ 100%	TBI	Neurological ICU	Increases cerebral aerobic metabolism.

[40], Vilalta et al.	Prospective cohort	30	FiO_2_ 100%	TBI	ICU	Improves brain redox state in patients with initially elevated brain lactate levels

[41], Diringer et al.	Prospective cohort	5	FiO_2_ 100%	TBI	Neurosurgical ICU	No improvement on brain metabolism.

[42], Puccio et al.	Prospective cohort	11	FiO_2_ 100%	TBI	Neurotrauma ICU	Brief periods do not produce oxidative stress and/or change antioxidant reserves in cerebrospinal fluid.

[43], Davis et al.	Retrospective cohort	3,420	PaO_2_> 64.9 kPa	TBI	-	Independently associated with increased mortality and decrease in good outcomes.

[44], Rincon et al.	Retrospective cohort	1,212	PaO_2_> 40.0 kPa	TBI	ICU	Independently associated with higher in-hospital mortality.

[45], Asher et al.	Retrospective cohort	193	PaO_2_> 64.8 kPa	TBI	-	Decrease in survival

[46], Raj et al.	Retrospective cohort	1,116	PaO_2_> 13.3 kPa	TBI	ICU	No effect on 6 month mortality

**Table 3:** Association between hyperoxemia and clinically relevant outcomes in sepsis.

Reference	Study design	Sample size	Hyperoxemia definition	Condition	Location	Conclusion
[19], Stolmeijer et al.	Prospective cohort	83	PaO_2_ > 13.5 kPa	Sepsis	ED	More than 64% of patients were hyperoxemic with 10 L O_2_/min. No association with mortality.

[47], Rossi et al.	Prospective cohort	14	FiO_2_ 100%	Sepsis	ICU	Decreases oxygen delivery in upper limbs.

[48], Pope et al.	Retrospective cohort	619	Central venous saturation (ScvO_2_) 90–100%	Sepsis	ED	Associated with increased mortality.

[49], Dahl et al.	Retrospective cohort	1,770	PaO_2_> 16.0 kPa	Sepsis	ICU	No effect on mortality, but hypoxemia and FiO_2_> 60% increased mortality.

## Abbreviations

CPR: Cardiopulmonary resuscitation
ED: Emergency department
ICU: Intensive care unit
MRI: Magnetic resonance imaging
NRM: Nonrebreathing mask
OHCA: Out-of-hospital cardiac arrest
PaO_2_: Partial pressure of oxygen in arterial blood
ROS: Reactive oxygen species
ROSC: Return of spontaneous circulation
ScvO_2_: Central venous oxygen saturation
STEMI: ST-segment elevation myocardial infarction
TBI: Traumatic brain injury
VF: Ventricular fibrillation
VM: VentiMask.

## Additional Points

*Strengths of This Study*. A comprehensive review about the association between hyperoxemia and clinically relevant outcomes in acutely ill adult patients.* Limitations of This Study*. Most reviewed studies are of a low level of evidence and mostly performed among patients admitted to the ICU, which does not cover the entire spectrum of emergency medicine.
